# Supplementary material for: Acute respiratory distress syndrome after SARS-CoV-2 infection on young adult population: International observational federated study based on electronic health records through the 4CE consortium
Source: PLoS One. 2023 Jan 4;18(1):e0266985. doi: 10.1371/journal.pone.0266985 (PMC9812312; doi:10.1371/journal.pone.0266985)
Supplement: S1 Appendix — (DOCX) [file pone.0266985.s001.docx]

**S1-Appendix: Medication class**

| **Medication Class** | **Class Name** | **Medication Name** | **ATC Code** |
| --- | --- | --- | --- |
| SIANES | ANESTHETICS, GENERAL | Ketamine | N01AX03 |
| SIANES | ANESTHETICS, GENERAL | Propofol | N01AX10 |
| SIANES | Benzodiazepine derivatives | Midazolam | N05CD08 |
| SIANES | MUSCLE RELAXANTS | Cisatracurium | M03AC11 |
| SIANES | MUSCLE RELAXANTS | rocuronium bromide | M03AC09 |
| SIANES | MUSCLE RELAXANTS | vecuronium | M03AC03 |
| SIANES | Other hypnotics and sedatives | Dexmetotomidine | N05CM18 |
| SICARDIAC | Adrenergic and dopaminergic agents | DOBUTamine | C01CA07 |
| SICARDIAC | Adrenergic and dopaminergic agents | Dopamine | C01CA04 |
| SICARDIAC | Adrenergic and dopaminergic agents | EPINEPHrine | C01CA24 |
| SICARDIAC | Adrenergic and dopaminergic agents | Norepinephrine | C01CA03 |
| SICARDIAC | Adrenergic and dopaminergic agents | phenylephrine | C01CA06 |
| SICARDIAC | Other cardiac stimulants | angiotensin II | C01CX09 |
| SICARDIAC | Other respiratory system products | Nitric Oxide | R07AX01 |
| SICARDIAC | Phosphodiesterase inhibitors | Milrinone | C01CE02 |
| SICARDIAC | Platelet aggregation inhibitors excl. heparin | Epoprostenol | B01AC09 |
| SICARDIAC | Vasopressin and analogues | Vasopressin | H01BA01 |
