# Supplementary material for: Acute respiratory distress syndrome after SARS-CoV-2 infection on young adult population: International observational federated study based on electronic health records through the 4CE consortium
Source: PLoS One. 2023 Jan 4;18(1):e0266985. doi: 10.1371/journal.pone.0266985 (PMC9812312; doi:10.1371/journal.pone.0266985)
Supplement: S2 Appendix — (DOCX) [file pone.0266985.s002.docx]

**S2-Appendix: Elixhauser comorbidities**

| **Elixhauser title** | **ICD10 code** | **ICD10 title** |
| --- | --- | --- |
| AIDS/HIV | B20 | Human immunodeficiency virus [HIV] disease |
| AIDS/HIV | B21 | Human immunodeficiency virus [HIV] disease resulting in malignant neoplasms |
| AIDS/HIV | B22 | Human immunodeficiency virus [HIV] disease resulting in other specified diseases |
| AIDS/HIV | B24 | Unspecified human immunodeficiency virus [HIV] disease |
| Alcohol abuse | F10 | Alcohol related disorders |
| Alcohol abuse | K70 | Alcoholic liver disease |
| Alcohol abuse | T51 | Toxic effect of alcohol |
| Cancer | C00 | Malignant neoplasm of lip |
| Cancer | C01 | Malignant neoplasm of base of tongue |
| Cancer | C02 | Malignant neoplasm of other and unspecified parts of tongue |
| Cancer | C03 | Malignant neoplasm of gum |
| Cancer | C04 | Malignant neoplasm of floor of mouth |
| Cancer | C05 | Malignant neoplasm of palate |
| Cancer | C06 | Malignant neoplasm of other and unspecified parts of mouth |
| Cancer | C07 | Malignant neoplasm of parotid gland |
| Cancer | C08 | Malignant neoplasm of other and unsp major salivary glands |
| Cancer | C09 | Malignant neoplasm of tonsil |
| Cancer | C10 | Malignant neoplasm of oropharynx |
| Cancer | C11 | Malignant neoplasm of nasopharynx |
| Cancer | C12 | Malignant neoplasm of pyriform sinus |
| Cancer | C13 | Malignant neoplasm of hypopharynx |
| Cancer | C14 | Malig neoplasm of sites in the lip, oral cavity and pharynx |
| Cancer | C15 | Malignant neoplasm of esophagus |
| Cancer | C16 | Malignant neoplasm of stomach |
| Cancer | C17 | Malignant neoplasm of small intestine |
| Cancer | C18 | Malignant neoplasm of colon |
| Cancer | C19 | Malignant neoplasm of rectosigmoid junction |
| Cancer | C20 | Malignant neoplasm of rectum |
| Cancer | C21 | Malignant neoplasm of anus and anal canal |
| Cancer | C22 | Malignant neoplasm of liver and intrahepatic bile ducts |
| Cancer | C23 | Malignant neoplasm of gallbladder |
| Cancer | C24 | Malignant neoplasm of other and unsp parts of biliary tract |
| Cancer | C25 | Malignant neoplasm of pancreas |
| Cancer | C26 | Malignant neoplasm of other and ill-defined digestive organs |
| Cancer | C30 | Malignant neoplasm of nasal cavity and middle ear |
| Cancer | C31 | Malignant neoplasm of accessory sinuses |
| Cancer | C32 | Malignant neoplasm of larynx |
| Cancer | C33 | Malignant neoplasm of trachea |
| Cancer | C34 | Malignant neoplasm of bronchus and lung |
| Cancer | C37 | Malignant neoplasm of thymus |
| Cancer | C38 | Malignant neoplasm of heart, mediastinum and pleura |
| Cancer | C39 | Malig neoplm of sites in the resp sys and intrathorac organs |
| Cancer | C40 | Malignant neoplasm of bone and articular cartilage of limbs |
| Cancer | C41 | Malignant neoplasm of bone/artic cartl of and unsp sites |
| Cancer | C43 | Malignant melanoma of skin |
| Cancer | C45 | Mesothelioma |
| Cancer | C46 | Kaposi's sarcoma |
| Cancer | C47 | Malignant neoplasm of prph nerves and autonomic nervous sys |
| Cancer | C48 | Malignant neoplasm of retroperitoneum and peritoneum |
| Cancer | C49 | Malignant neoplasm of other connective and soft tissue |
| Cancer | C50 | Malignant neoplasm of breast |
| Cancer | C51 | Malignant neoplasm of vulva |
| Cancer | C52 | Malignant neoplasm of vagina |
| Cancer | C53 | Malignant neoplasm of cervix uteri |
| Cancer | C54 | Malignant neoplasm of corpus uteri |
| Cancer | C55 | Malignant neoplasm of uterus, part unspecified |
| Cancer | C56 | Malignant neoplasm of ovary |
| Cancer | C57 | Malignant neoplasm of other and unsp female genital organs |
| Cancer | C58 | Malignant neoplasm of placenta |
| Cancer | C60 | Malignant neoplasm of penis |
| Cancer | C61 | Malignant neoplasm of prostate |
| Cancer | C62 | Malignant neoplasm of testis |
| Cancer | C63 | Malignant neoplasm of other and unsp male genital organs |
| Cancer | C64 | Malignant neoplasm of kidney, except renal pelvis |
| Cancer | C65 | Malignant neoplasm of renal pelvis |
| Cancer | C66 | Malignant neoplasm of ureter |
| Cancer | C67 | Malignant neoplasm of bladder |
| Cancer | C68 | Malignant neoplasm of other and unspecified urinary organs |
| Cancer | C69 | Malignant neoplasm of eye and adnexa |
| Cancer | C70 | Malignant neoplasm of meninges |
| Cancer | C71 | Malignant neoplasm of brain |
| Cancer | C72 | Malig neoplm of spinal cord, cranial nerves and oth prt cnsl |
| Cancer | C73 | Malignant neoplasm of thyroid gland |
| Cancer | C74 | Malignant neoplasm of adrenal gland |
| Cancer | C75 | Malignant neoplasm of endo glands and related structures |
| Cancer | C76 | Malignant neoplasm of other and ill-defined sites |
| Cancer | C77 | Secondary and unspecified malignant neoplasm of lymph nodes |
| Cancer | C78 | Secondary malignant neoplasm of resp and digestive organs |
| Cancer | C79 | Secondary malignant neoplasm of other and unspecified sites |
| Cancer | C80 | Malignant neoplasm without specification of site |
| Cancer | C81 | Hodgkin lymphoma |
| Cancer | C82 | Follicular lymphoma |
| Cancer | C83 | Non-follicular lymphoma |
| Cancer | C84 | Mature T/NK-cell lymphomas |
| Cancer | C85 | Oth and unspecified types of non-Hodgkin lymphoma |
| Cancer | C88 | Malig immunoproliferative dis and certain oth B-cell lymph |
| Cancer | C90 | Multiple myeloma and malignant plasma cell neoplasms |
| Cancer | C96 | Oth & unsp malig neoplm of lymphoid, hematpoetc and rel tiss |
| Cancer | C97 | Malignant neoplasms of independent (primary) multiple sites |
| Chronic pulmonary disease | I27 | Other pulmonary heart diseases |
| Chronic pulmonary disease | J40 | Bronchitis, not specified as acute or chronic |
| Chronic pulmonary disease | J41 | Simple and mucopurulent chronic bronchitis |
| Chronic pulmonary disease | J42 | Unspecified chronic bronchitis |
| Chronic pulmonary disease | J43 | Emphysema |
| Chronic pulmonary disease | J44 | Other chronic obstructive pulmonary disease |
| Chronic pulmonary disease | J45 | Asthma |
| Chronic pulmonary disease | J46 | Status asthmaticus |
| Chronic pulmonary disease | J47 | Bronchiectasis |
| Chronic pulmonary disease | J60 | Coalworker's pneumoconiosis |
| Chronic pulmonary disease | J61 | Pneumoconiosis due to asbestos and other mineral fibers |
| Chronic pulmonary disease | J62 | Pneumoconiosis due to dust containing silica |
| Chronic pulmonary disease | J63 | Pneumoconiosis due to other inorganic dusts |
| Chronic pulmonary disease | J64 | Unspecified pneumoconiosis |
| Chronic pulmonary disease | J65 | Pneumoconiosis associated with tuberculosis |
| Chronic pulmonary disease | J66 | Airway disease due to specific organic dust |
| Chronic pulmonary disease | J67 | Hypersensitivity pneumonitis due to organic dust |
| Chronic pulmonary disease | J68 | Resp cond d/t inhalation of chemicals, gas, fumes and vapors |
| Chronic pulmonary disease | J70 | Respiratory conditions due to other external agents |
| Congestive heart failure | I09 | Other rheumatic heart diseases |
| Congestive heart failure | I11 | Hypertensive heart disease |
| Congestive heart failure | I13 | Hypertensive heart and chronic kidney disease |
| Congestive heart failure | I25 | Chronic ischemic heart disease |
| Congestive heart failure | I42 | Cardiomyopathy |
| Congestive heart failure | I43 | Cardiomyopathy in diseases classified elsewhere |
| Congestive heart failure | I50 | Heart failure |
| Congestive heart failure | P29 | Cardiovascular disorders originating in the perinatal period |
| Diabetes | E10 | Type 1 diabetes mellitus |
| Diabetes | E11 | Type 2 diabetes mellitus |
| Diabetes | E12 | Malnutrition-related diabetes mellitus |
| Diabetes | E13 | Other specified diabetes mellitus |
| Diabetes | E14 | Unspecified diabetes mellitus |
| Drug abuse | F11 | Opioid related disorders |
| Drug abuse | F12 | Cannabis related disorders |
| Drug abuse | F13 | Sedative, hypnotic, or anxiolytic related disorders |
| Drug abuse | F14 | Cocaine related disorders |
| Drug abuse | F15 | Other stimulant related disorders |
| Drug abuse | F16 | Hallucinogen related disorders |
| Drug abuse | F18 | Inhalant related disorders |
| Drug abuse | F19 | Other psychoactive substance related disorders |
| Hypertension | I10 | Essential (primary) hypertension |
| Hypertension | I11 | Hypertensive heart disease |
| Hypertension | I12 | Hypertensive chronic kidney disease |
| Hypertension | I13 | Hypertensive heart and chronic kidney disease |
| Hypertension | I15 | Secondary hypertension |
| Hypothyroidism | E00 | Congenital iodine-deficiency syndrome |
| Hypothyroidism | E01 | Iodine-deficiency related thyroid disorders and allied cond |
| Hypothyroidism | E02 | Subclinical iodine-deficiency hypothyroidism |
| Hypothyroidism | E03 | Other hypothyroidism |
| Hypothyroidism | E89 | Postproc endocrine and metabolic comp and disorders, NEC |
| Liver disease | B18 | Chronic viral hepatitis |
| Liver disease | I85 | Esophageal varices |
| Liver disease | K70 | Alcoholic liver disease |
| Liver disease | K71 | Toxic liver disease |
| Liver disease | K72 | Hepatic failure, not elsewhere classified |
| Liver disease | K73 | Chronic hepatitis, not elsewhere classified |
| Liver disease | K74 | Fibrosis and cirrhosis of liver |
| Liver disease | K76 | Other diseases of liver |
| Obesity | E66 | Overweight and obesity |
| Paralysis | G04 | Encephalitis, myelitis and encephalomyelitis |
| Paralysis | G11 | Hereditary ataxia |
| Paralysis | G80 | Cerebral palsy |
| Paralysis | G81 | Hemiplegia and hemiparesis |
| Paralysis | G82 | Paraplegia (paraparesis) and quadriplegia (quadriparesis) |
| Paralysis | G83 | Other paralytic syndromes |
| Peptic ulcer disease | K25 | Gastric ulcer |
| Peptic ulcer disease | K26 | Duodenal ulcer |
| Peptic ulcer disease | K27 | Peptic ulcer, site unspecified |
| Peptic ulcer disease | K28 | Gastrojejunal ulcer |
| Peripheral vascular disease | I70 | Atherosclerosis |
| Peripheral vascular disease | I71 | Aortic aneurysm and dissection |
| Peripheral vascular disease | I73 | Other peripheral vascular diseases |
| Peripheral vascular disease | I77 | Other disorders of arteries and arterioles |
| Peripheral vascular disease | I79 | Disord of art, arterioles and capilare in dis classd elswhr |
| Peripheral vascular disease | K55 | Vascular disorders of intestine |
| Psychoses | F20 | Schizophrenia |
| Psychoses | F22 | Delusional disorders |
| Psychoses | F23 | Brief psychotic disorder |
| Psychoses | F24 | Shared psychotic disorder |
| Psychoses | F25 | Schizoaffective disorders |
| Psychoses | F28 | Oth psych disorder not due to a sub or known physiol cond |
| Psychoses | F29 | Unsp psychosis not due to a substance or known physiol cond |
| Psychoses | F30 | Manic episode |
| Psychoses | F31 | Bipolar disorder |
| Renal failure | I12 | Hypertensive chronic kidney disease |
| Renal failure | I13 | Hypertensive heart and chronic kidney disease |
| Renal failure | N18 | Chronic kidney disease (CKD) |
| Renal failure | N19 | Unspecified kidney failure |
| Renal failure | N25 | Disorders resulting from impaired renal tubular function |
| Renal failure | Z49 | Encounter for care involving renal dialysis |
| Valvular disease | A52 | Late syphilis |
| Valvular disease | I05 | Rheumatic mitral valve diseases |
| Valvular disease | I06 | Rheumatic aortic valve diseases |
| Valvular disease | I07 | Rheumatic tricuspid valve diseases |
| Valvular disease | I08 | Multiple valve diseases |
| Valvular disease | I09 | Other rheumatic heart diseases |
| Valvular disease | I34 | Nonrheumatic mitral valve disorders |
| Valvular disease | I35 | Nonrheumatic aortic valve disorders |
| Valvular disease | I36 | Nonrheumatic tricuspid valve disorders |
| Valvular disease | I37 | Nonrheumatic pulmonary valve disorders |
| Valvular disease | I38 | Endocarditis, valve unspecified |
| Valvular disease | I39 | Endocarditis and heart valve disorder in diseases classified elsewhere |
| Valvular disease | Q23 | Congenital malformations of aortic and mitral valves |
| Valvular disease | Z95 | Presence of cardiac and vascular implants and grafts |
