# Supplementary material for: Acute respiratory distress syndrome after SARS-CoV-2 infection on young adult population: International observational federated study based on electronic health records through the 4CE consortium
Source: PLoS One. 2023 Jan 4;18(1):e0266985. doi: 10.1371/journal.pone.0266985 (PMC9812312; doi:10.1371/journal.pone.0266985)
Supplement: S3 Appendix — (DOCX) [file pone.0266985.s003.docx]

**S3-Appendix: Complication classification**

| **Complication class** | **ICD10 code** | **ICD10 title** |
| --- | --- | --- |
| Acute kidney failure | N17 | Acute kidney failure |
| Cardiac arrest | I46 | Cardiac arrest |
| Cardiac complication | I21 | ST elevation (STEMI) and non-ST elevation (NSTEMI) myocardial infarction |
| Cardiac complication | I22 | Subsequent ST elevation (STEMI) and non-ST elevation (NSTEMI) myocardial infarction |
| Cardiac complication | I24 | Other acute ischemic heart diseases |
| Cardiac complication | I27 | Other pulmonary heart diseases |
| Cardiac complication | I30 | Acute pericarditis |
| Cardiac complication | I31 | Other diseases of pericardium |
| Cardiac complication | I32 | Pericarditis in diseases classified elsewhere |
| Cardiac complication | I33 | Acute and subacute endocarditis |
| Cardiac complication | I40 | Acute myocarditis |
| Cardiac complication | I42 | Cardiomyopathy |
| Cardiac complication | I50 | Heart failure |
| Cardiac complication | I51 | Complications and ill-defined descriptions of heart disease |
| Cardiac Rhythm/conduction disorder | I44 | Atrioventricular and left bundle-branch block |
| Cardiac Rhythm/conduction disorder | I45 | Other conduction disorders |
| Cardiac Rhythm/conduction disorder | I46 | Cardiac arrest |
| Cardiac Rhythm/conduction disorder | I47 | Paroxysmal tachycardia |
| Cardiac Rhythm/conduction disorder | I48 | Atrial fibrillation and flutter |
| Cardiac Rhythm/conduction disorder | I49 | Other cardiac arrhythmias |
| Digestive complication | K21 | Gastro-esophageal reflux disease |
| Digestive complication | K22 | Other diseases of esophagus |
| Digestive complication | K25 | Gastric ulcer |
| Digestive complication | K26 | Duodenal ulcer |
| Digestive complication | K27 | Peptic ulcer, site unspecified |
| Digestive complication | K28 | Gastrojejunal ulcer |
| Digestive complication | K29 | Gastritis and duodenitis |
| Digestive complication | K30 | Functional dyspepsia |
| Digestive complication | K31 | Other diseases of stomach and duodenum |
| Digestive complication | K56 | Paralytic ileus and intestinal obstruction without hernia |
| Digestive complication | R10 | Abdominal and pelvic pain |
| Digestive complication | R11 | Nausea and vomiting |
| Digestive complication | R13 | Aphagia and dysphagia |
| Digestive complication | R16 | Hepatomegaly and splenomegaly, not elsewhere classified |
| Disorders of fluid, electrolyte, and acid-base balance | E87 | Other disorders of fluid, electrolyte and acid-base balance |
| Haematological disorder | D59 | Acquired hemolytic anemia |
| Haematological disorder | D62 | Acute posthemorrhagic anemia |
| Haematological disorder | D65 | Disseminated intravascular coagulation [defibrination syndrome] |
| Haematological disorder | D68 | Other coagulation defects |
| Haematological disorder | D69 | Purpura and other hemorrhagic conditions |
| Haematological disorder | D70 | Neutropenia |
| Haematological disorder | D72 | Other disorders of white blood cells |
| Hemodynamic disorder | E86 | Volume depletion |
| Hemodynamic disorder | I95 | Hypotension |
| Hemodynamic disorder | R03 | Abnormal blood-pressure reading, without diagnosis |
| Arterial embolism and thrombosis | I74 | Arterial embolism and thrombosis |
| Stroke | I62 | Other and unspecified nontraumatic intracranial hemorrhage |
| Stroke | I63 | Cerebral infarction |
| Stroke | I65 | Occlusion and stenosis of precerebral arteries, not resulting in cerebral infarction |
| Stroke | I66 | Occlusion and stenosis of cerebral arteries, not resulting in cerebral infarction |
| Stroke | I67 | Other cerebrovascular diseases |
| Phlebitis and thrombophlebitis | I80 | Phlebitis and thrombophlebitis |
| Phlebitis and thrombophlebitis | I81 | Portal vein thrombosis |
| Phlebitis and thrombophlebitis | I82 | Other venous embolism and thrombosis |
| Pulmonary embolism | I26 | Pulmonary embolism |
| Respiratory complication (excluding ARDS) | J20 | Acute bronchitis |
| Respiratory complication (excluding ARDS) | J69 | Pneumonitis due to solids and liquids |
| Respiratory complication (excluding ARDS) | J81 | Pulmonary edema |
| Respiratory complication (excluding ARDS) | J84 | Other interstitial pulmonary diseases |
| Respiratory complication (excluding ARDS) | J85 | Abscess of lung and mediastinum |
| Respiratory complication (excluding ARDS) | J90 | Pleural effusion, not elsewhere classified |
| Respiratory complication (excluding ARDS) | J91 | Pleural effusion in conditions classified elsewhere |
| Respiratory complication (excluding ARDS) | J95 | Intraoperative and postprocedural complications and disorders of respiratory system, not elsewhere classified |
| Respiratory complication (excluding ARDS) | J96 | Respiratory failure, not elsewhere classified |
| Respiratory complication (excluding ARDS) | J98 | Other respiratory disorders |
| Pressure ulcer | L89 | Pressure ulcer |
| Viral reactivation | B02 | Zoster [herpes zoster] |
| Viral reactivation | B00 | Herpesviral [herpes simplex] infections |
| Aspergillosis | B44 | Aspergillosis |
| Candidiasis | B37 | Candidiasis |
| Candidiasis | B36 | Other superficial mycoses |
| Candidiasis | B49 | Unspecified mycosis |
| Other fungal infection | B36 | Other superficial mycoses |
| Other fungal infection | B49 | Unspecified mycosis |
| Bacterial infection | A41 | Other sepsis |
| Bacterial infection | A46 | Erysipelas |
| Bacterial infection | A49 | Bacterial infection of unspecified site |
| Bacterial infection | B95 | Streptococcus, Staphylococcus, and Enterococcus as the cause of diseases classified elsewhere |
| Bacterial infection | B96 | Other bacterial agents as the cause of diseases classified elsewhere |
| Bacterial intestinal infection | A04 | Other bacterial intestinal infections |
| Bacterial intestinal infection | A09 | Infectious gastroenteritis and colitis, unspecified |
| Respiratory bacterial superinfection | J14 | Pneumonia due to Hemophilus influenzae |
| Respiratory bacterial superinfection | J15 | Bacterial pneumonia, not elsewhere classified |
| Respiratory bacterial superinfection | J18 | Pneumonia, unspecified organism |
| Pneumonia due to Streptococcus pneumoniae | J13 | Pneumonia due to Streptococcus pneumoniae |
| Streptococcal sepsis | A40 | Streptococcal sepsis |
