# Supplementary material for: Acute respiratory distress syndrome after SARS-CoV-2 infection on young adult population: International observational federated study based on electronic health records through the 4CE consortium
Source: PLoS One. 2023 Jan 4;18(1):e0266985. doi: 10.1371/journal.pone.0266985 (PMC9812312; doi:10.1371/journal.pone.0266985)
Supplement: S1 Table — (DOCX) [file pone.0266985.s004.docx]

**S1-Table:** Number and percentage of patients per age groups, per sex, per Elixhauser comorbidities for all groups

|  | **ARDS** | | **NO SEVERE** | | **SEVERE NO ARDS** | |
| --- | --- | --- | --- | --- | --- | --- |
|  | **18-49** | **>49** | **18-49** | **>49** | **18-49** | **>49** |
|  | **n (%)** | **n (%)** | **n (%)** | **n (%)** | **n (%)** | **n (%)** |
|  |  |  |  |  |  |  |
| **Number of patient (% on total)** | 1001 (1.3) | 6378 (8.5) | 10107 (13.4) | 46512 (61.7) | 1674 (2,2) | 9705 (12,9) |
|  |  |  |  |  |  |  |
| **Age groups** |  |  |  |  |  |  |
| 18to25 | 43 (4.3) | 0 (0) | 1207 (11.9) | 0 (0) | 244 (14.6) | 0 (0) |
| 26to49 | 966 (96.5) | 0 (0) | 8900 (88.1) | 0 (0) | 1433 (85.6) | 0 (0) |
| 50to69 | 0 (0) | 3367 (52.8) | 0 (0) | 19471 (41.9) | 0 (0) | 3855 (39.7) |
| 70to79 | 0 (0) | 2073 (32.5) | 0 (0) | 14506 (31.2) | 0 (0) | 3247 (33.5) |
| 80plus | 0 (0) | 938 (14.7) | 0 (0) | 12535 (27) | 0 (0) | 2607 (26.9) |
|  |  |  |  |  |  |  |
| **Sex** |  |  |  |  |  |  |
| female | 327 (32.7) | 1581 (24.8) | 4427 (43.8) | 12918 (27.8) | 873 (52.2) | 2848 (29.3) |
| male | 672 (67.1) | 4797 (75.2) | 5680 (56.2) | 33593 (72.2) | 803 (48) | 6861 (70.7) |
|  |  |  |  |  |  |  |
| **Previous visits with HS, defined by at least one ICD code from -365 days to + 14 days before admission** | | | | | | |
| no previous contact | 681 (68) | 3181 (49.9) | 4369 (43.2) | 13108 (28.2) | 471 (28.1) | 2245 (23.1) |
| previous contact | 312 (31.2) | 3197 (50.1) | 5738 (56.8) | 33404 (71.8) | 1197 (71.5) | 7464 (76.9) |
|  |  |  |  |  |  |  |
| **Comorbidities (Elix Hauser class), ICD code from -365 days before to + 90 days after admission** | | | | | |  |
| AIDS/HIV | 12 (1.2) | 81 (1.3) | 121 (1.2) | 424 (0.9) | 21 (1.3) | 63 (0.6) |
| Alcohol abuse | 59 (5.9) | 327 (5.1) | 895 (8.9) | 3743 (8) | 142 (8.5) | 715 (7.4) |
| Cancer | 37 (3.7) | 688 (10.8) | 280 (2.8) | 6518 (14) | 132 (7.9) | 1770 (18.2) |
| Chronic pulmonary disease | 219 (21.9) | 2009 (31.5) | 1406 (13.9) | 13791 (29.7) | 339 (20.3) | 3709 (38.2) |
| Congestive heart failure | 143 (14.3) | 2335 (36.6) | 532 (5.3) | 17404 (37.4) | 238 (14.2) | 4745 (48.9) |
| Diabetes | 322 (32.2) | 3065 (48.1) | 1691 (16.7) | 19375 (41.7) | 326 (19.5) | 4578 (47.2) |
| Drug abuse | 65 (6.5) | 176 (2.8) | 828 (8.2) | 2393 (5.1) | 141 (8.4) | 459 (4.7) |
| Hypertension | 382 (38.2) | 4534 (71.1) | 2274 (22.5) | 32747 (70.4) | 522 (31.2) | 7301 (75.2) |
| Hypothyroidism | 45 (4.5) | 737 (11.6) | 431 (4.3) | 5928 (12.7) | 100 (6) | 1447 (14.9) |
| Liver disease | 179 (17.9) | 922 (14.5) | 960 (9.5) | 4627 (9.9) | 232 (13.9) | 1317 (13.6) |
| Obesity | 533 (53.2) | 2366 (37.1) | 2759 (27.3) | 11302 (24.3) | 456 (27.2) | 2758 (28.4) |
| Paralysis | 64 (6.4) | 360 (5.6) | 162 (1.6) | 1473 (3.2) | 73 (4.4) | 456 (4.7) |
| Peptic ulcer disease | 36 (3.6) | 183 (2.9) | 60 (0.6) | 869 (1.9) | 31 (1.9) | 318 (3.3) |
| Peripheral vascular disease | 37 (3.7) | 853 (13.4) | 184 (1.8) | 6561 (14.1) | 109 (6.5) | 1868 (19.2) |
| Psychoses | 52 (5.2) | 270 (4.2) | 599 (5.9) | 2831 (6.1) | 97 (5.8) | 566 (5.8) |
| Renal failure | 131 (13.1) | 1795 (28.1) | 590 (5.8) | 12446 (26.8) | 222 (13.3) | 3566 (36.7) |
| Valvular disease | 92 (9.2) | 1337 (21) | 346 (3.4) | 10245 (22) | 169 (10.1) | 2938 (30.3) |
|  |  |  |  |  |  |  |
| **Mortality at 90 days** | 162 (16.2) | 2619 (41.1) | 24 (0.2) | 2802 (6.0) | 92 (5.5) | 2180 (22.5) |
