# Supplementary material for: Acute respiratory distress syndrome after SARS-CoV-2 infection on young adult population: International observational federated study based on electronic health records through the 4CE consortium
Source: PLoS One. 2023 Jan 4;18(1):e0266985. doi: 10.1371/journal.pone.0266985 (PMC9812312; doi:10.1371/journal.pone.0266985)
Supplement: S2 Table — Risk ratio associated in uni-variable analysis for the sub population which had previous hospital visits and considering only the ICD code related to those previous visits (one year and– 14 before the admission). (DOCX) [file pone.0266985.s005.docx]

**S2-Table:** sub population which had previous hospital visits and considering only the ICD code related to those previous visits (one year and – 14 before the admission). Number and percentage of patients per Elixhauser comorbidities for young adult patients with ARDS and non severe young adult patients. Risk ratio associated in uni-variable analysis.

|  | **ICD code from -365 days to -14 days before admission** | | | |
| --- | --- | --- | --- | --- |
| **Elix Hauser classes** | **ARDS** | **NO SEVERE** | **Univariate analysis** | |
|  | **ages 18-49** | **ages 18-49** |  |  |
|  | **n = 312** | **n = 5738** | **Risk Ratio with CI (95%)** | **p-value** |
|  | **n (%)** | **n (%)** |  |  |
| AIDS/HIV | 0 (0) | 63 (1.1) | -- | -- |
| Alcohol abuse | 16 (5.1) | 560 (9.8) | 1 [0.6;1.9] | 0.924 |
| Cancer | 30 (9.6) | 191 (3.3) | 2.2 [1.5;3.3] | <0.001 |
| Chronic pulmonary disease | 36 (11.5) | 670 (11.7) | 1.3 [0.9;1.7] | 0.166 |
| Congestive heart failure | 45 (14.4) | 266 (4.6) | 3.3 [2.5;4.5] | <0.001 |
| Diabetes | 75 (24) | 879 (15.3) | 1.9 [1.4;2.5] | <0.001 |
| Drug abuse | 11 (3.5) | 500 (8.7) | 0.7 [0.4;1.1] | 0.108 |
| Hypertension | 105 (33.7) | 1321 (23) | 2.1 [1.5;2.8] | <0.001 |
| Hypothyroidism | 29 (9.3) | 259 (4.5) | 2.2 [1.1;4.2] | 0.019 |
| Liver disease | 42 (13.5) | 430 (7.5) | 1.6 [1.1;2.4] | 0.009 |
| Obesity | 95 (30.4) | 1160 (20.2) | 1.8 [1.3;2.5] | 0.001 |
| Paralysis | 22 (7.1) | 79 (1.4) | 3.5 [2.2;5.4] | <0.001 |
| Peptic ulcer disease | 6 (1.9) | 29 (0.5) | 6 [3.2;11.6] | <0.001 |
| Peripheral vascular disease | 24 (7.7) | 110 (1.9) | 2.5 [1.5;4.2] | <0.001 |
| Psychoses | 10 (3.2) | 360 (6.3) | 0.8 [0.5;1.3] | 0.398 |
| Renal failure | 50 (16) | 348 (6.1) | 2.7 [2;3.7] | <0.001 |
| Valvular disease | 26 (8.3) | 171 (3) | 3 [1.9;4.8] | <0.001 |
