# Supplementary material for: Acute respiratory distress syndrome after SARS-CoV-2 infection on young adult population: International observational federated study based on electronic health records through the 4CE consortium
Source: PLoS One. 2023 Jan 4;18(1):e0266985. doi: 10.1371/journal.pone.0266985 (PMC9812312; doi:10.1371/journal.pone.0266985)
Supplement: Attachment — Submitted filename: Response_to_reviewer.pdf [file pone.0266985.s006.pdf]

Response to reviewer

RE: entitled Acute respiratory distress syndrome after SARS-CoV-2 infection on young adult population: international observational study based on electronic health records through the 4CE consortium

Dear Editor and Reviewer,

Thank you very much for your comments and constructive criticisms targeted at improving our manuscript submission. Below you will find our responses to the comments. The manuscript has also been modified accordingly. The novelty of the methods has been emphasized and additional descriptive data have been generated.

The main strength of this work is the very large dataset from which the cohort was extracted. But in its current form, priority for CCM will be limited. A few important concerns are noted including:

1. Dichotomization of the continuous variable age leads to loss of information, and creates counterintuitive relationships. In this case, someone who is 50 is considered old, while someone who is 49 is considered young. The 50 year-old is also considered just as old as a 90 year-old.

Through the 4CE consortium, the age is not collected as a numeric value but by age groups (18−25, 26−49, 50−69, 70−79, and 80+ years old), as described in the method section. It is an intrinsic limit of our analysis, but this choice has been made to bring more security/de-identification to the data collection process which permits an easier regulatory process for international aggregated data sharing.

To highlight this limit, we have added in the discussion the following sentences:

> "More detailed analysis on age's threshold was not possible because age was intentionally not collected by the 4CE consortium as a continuous variable. This choice was made to ensure greater security/de-identification on the data collection process which allowed for an easier regulatory process for international aggregated data sharing"

2. As the authors note, the use of ICD codes alone is a limitation, especially in this multi-institutional dataset where coding practices may vary. The coding for ARDS may lack both sensitivity and specificity, and the temporal relationships cannot be inferred. This

may explain why things like peptic ulcer disease and paralysis were identified as risk factors. These seem just as likely to be sequelae of COVID ARDS as they are risk factors.

The use of ICD code for the ARDS detection is a limitation of our analysis. However, because billing codes are related to reimbursement in most countries and ARDS associated with higher levels of care, a correct sensibility is expected.

The following sentence was added to the limits section:

> “For the detection of ARDS patients, a correct sensibility is expected as billing code is related to reimbursement in most countries and ARDS associated with heavy care.”

Regarding the temporal relationships, patients included in our analysis had positive reverse transcription PCR test for SARS-CoV-2 infection 7 days before to 14 days after the date of admission. Those inclusion criteria were fixed by the 4CE consortium, for all the studies. This inclusion criteria allows us to ensure that the patient had the COVID-19 infection at least at the beginning of the hospitalization. Whereas it is not possible to establish a clear temporal relationship between ARDS and COVID-19 with ICD codes, it would be extremely rare that the development of ARDS during the hospitalization of a COVID positive patient had no relation with COVID-19 infection. It is possible that COVID infection was not the primary cause of the ARDS but most likely had an impact on the ARDS development.

As explained in the discussion, one limitation of relying on billing codes to identify comorbidities is the challenge of accurately distinguishing comorbidities from complications.

In the main analysis, comorbidities were considered as those diagnoses from billing codes assigned up to one year before and up to 90 days after the admission. This approach is more sensitive, but it can lead to considering complications as comorbidities. It is particularly true for peptic ulcer disease, which was identified as a comorbidity associated with ARDS, but which is also known to be a common complication of mechanical ventilation(32, 33).

To distinguish between comorbidities and complications, we performed a complementary univariable analysis on the subpopulation which had previous hospital visits and by considering only the ICD code related to those previous visits (one year and – 14 days before the admission). This subpopulation represents 31% (312 patients) of the young ARDS and 57% (5738 patients) of the young patient without ARDS.

In this univariable analysis, ARDS was associated with the presence of peptic ulcer disease in a previous hospitalization. It was also true for paralysis.

We added this sub-analysis in the appendix, but we did not present it in the main analysis because only a third of the ARDS patients had a previous hospitalization.

To address this comment more clearly, we add the following sentence in the discussion

> "In our analysis, comorbidities were considered as those diagnoses from billing codes assigned up to one year before and up to 90 days after the admission. This approach is more sensitive, but it can lead to considering complications as comorbidities. It is particularly true for peptic ulcer disease or paralysis which was identified as a comorbidity associated with ARDS but which is also known to be a common complication of mechanical ventilation(32, 33) or prolonged ICU admission. To distinguish between comorbidities and complications, we perform a complementary univariable analysis on the sub-population who had previous hospital visits and by considering only the ICD code related to those previous visits (one year to 14 days before the admission). In this univariable analysis presented in appendix 4, ARDS was associated with the presence in a previous hospitalization of peptic ulcer disease or paralysis, which explained our choice of considering them as comorbidities. "

3. The multivariable modelling would benefit from some additional explanation. Why were these particular variables chosen? The fact that hypertension was not identified as a risk factor leads to questions about the choice of confounders and methods used.

Firstly univariable analysis was performed at each HS (health system). Those univariable analyses were aggregated through a random-effect meta-analysis to account for heterogeneity between health care systems. Comorbidities significantly associated with ARDS in this aggregated univariable analysis were selected for the multivariable analysis. Multivariable analysis was performed at each HS and then aggregated through another meta-analysis with random effect.

The following text was added to the method discussion

> "First univariable analysis was performed at each HS and aggregated through a random-effect meta-analysis to account for heterogeneity between health care systems. In addition, comorbidities associated with ARDS in this meta univariable analysis, and sex were selected for a multivariable analysis. Multivariable analysis was performed at each HS and then aggregated through another meta-analysis with random effect."

As mentioned, Hypertension was not significantly associated with ARDS in our multivariable analysis (RR : 1.36 [0.98; 1.89], p = 0.062) despite its association with poor outcomes in several cohorts of COVID-19 patients(2, 15). We agree that the choice of the confounding factor could explain why the p-value is not below the threshold 0.05.

To take this comment into consideration, we add the following in the discussion

> “Hypertension was not significantly associated with ARDS in our study, possibly due to the choice of the variable include in the multivariable analysis or/and a lack of power “

4. Death as a competing risk in identifying complications -- eg., younger patients had a higher risk of developing Strep pneumonia as a complication, but is this simply a reflection of the fact that the older patients didn't survive long enough to develop VAP?

We agree that death for older adult is a competing risk, and we add this comment in our discussion

> “The higher risk of developing pneumonia due to Streptococcus pneumoniae and Streptococcal sepsis in young adults is probably related to their greater survival rate compared to older patients which give them the opportunity to develop those complications.”

5. Many of these findings lack novelty, eg. the association between severe COVID and risk factors like obesity and diabetes.

As mentioned in the introduction, several studies have investigated the comorbidities associated with severe COVID-19 infection in the general population with a national and international population. Severe definition varies between studies (death, ICU, …). Few national studies have investigated the young adult population, and, to our knowledge, none were international, focused only on ARDS patients, and included such a large population. In addition to the method, we believe that those aspects add to the novelty of our analysis.

In parallel, we understand that those results could appear lacking in novelty, but from our understanding, they reflect more that the comorbidities associated with ARDS in young adults are similar than the ones associated to the general population to severe COVID.

To more clearly describe those aspects, the following sentence was added to the discussion:

> “Though our analysis, it seems that most of the comorbidities associated with ARDS on the young adult population are the same than the ones associated with poor outcomes after SARS-CoV-2 infection in the general population

6. We observed that patients developing ARDS in this young adult population had a high prevalence of obesity, diabetes, and hypertension. From the analysis it appears that hypertension was not in fact associated.

As mentioned, hypertension is not significantly associated with the development of ARDS in our analysis. However, the hypertension prevalence (38%) in the young ARDS population is high comparing to the prevalence of hypertension in general in young adult (7-18 %)[1]

To remove all ambiguities, we change the related sentence as follow in the abstract

> “In this population, we described a high prevalence of obesity (53%), hypertension (38%- although not significantly associated with ARDS) and diabetes (32%).”

And in the conclusion

> “Young adults developing ARDS presented a high prevalence of comorbidities, particularly obesity, hypertension (although not being associated with ARDS) and diabetes”

7. Why were SEVERE_NO_ARDS cases excluded? There may be biases introduced from this.

As noticed, bias of selection could have been introduced because patients with mechanical ventilation, sedatives/anesthetics or treatment for shock but without an ARDS code (SEVERE_NO_ARDS) were not included to identify comorbidities associated with ARDS.

The choice, to not include them in this comparison, was conducted to eliminate potential miscoded ARDS patients and patients with severe disease or care not related to SARS-CoV-2 infection but with a concomitant infection.

Those patients could have been included in the ARDS population, but the objective of this study was to focus precisely on ARDS patients, and this grouping would have resulted in

---

[1] https://www.ahajournals.org/doi/full/10.1161/HYPERTENSIONAHA.119.13820

a significant measurement bias. Especially considering the fact that the young SEVERE_NO_ARDS patients number is greater than the one from ARDS patients.

Another option would have been to group those patients with the NO_SEVERE patients, but this option would also result from a significant measurement bias. Miscoded ARDS patients could have hidden relevant associations and/or patients with severe disease or care not related to SARS-CoV-2 infection but with a concomitant infection could have created a non relevant association.

In addition, we believe that the descriptive analysis of the SEVERE_NO_ARDS brings credits to this choice (appendix 5). Indeed, the young SEVERE_NO_ARDS population had a higher percentage of women (SEVERE_NO_ARDS: 52%, ARDS : 33%, No SEVERE: 44%). They also had a higher percentage of patients with previous contact with the healthcare system (SEVERE_NO_ARDS: 72%, ARDS : 31%, No SEVERE: 57%). Moreover, 15.1% of the SEVERE_NO_ARDS patients had a billing code associated with pregnancy, 36.1% with long-term drug therapy. These results suggest that the COVID-19 infection was simply concomitant but not the main cause of these hospitalizations.

However, to highlight this potential bias the following sentences was added to the limits section:

> “Patients with mechanical ventilation, sedatives/anesthetics or treatment for shock but without ARDS code were not included, which could generate a selection bias. This choice was conducted to eliminate potential miscoded ARDS patients and patients with severe disease or care not related to SARS-CoV-2 infection but with a concomitant infection. In addition, we believe that the descriptive analysis of the SEVERE_NO_ARDS brings credits to this choice (appendix 5). Compared to the other groups, SEVERE_NO_ARDS population had the higher percentage of women (52.2%) and of patients with previous contact with the healthcare system (72%). In addition, 15.1% of those patients had a billing code associated with pregnancy and 36.1% with a long-term drug therapy. These results suggest that the COVID-19 infection was simply concomitant but not the main cause of these hospitalizations”

8. The large tables are somewhat cumbersome and difficult to navigate.

The number of tables and figures is limited by the journal policy which explains the large cumbersome tables. However, to make them easier to read we keep only one number after the comma, when possible.

9. Guidance on causal inference modelling in critical care studies can be found in PMID30230362.

Thank you very much for sharing this article. Our objective with our multivariable analysis was not to establish the causal inference between ARDS and certain comorbidities. Our objective was simply to highlight populations with a higher risk of developing ARDS in young adults and bring more knowledge on this specific young adult population. Considering it, we believe that our approach and methodology for multivariable analysis is acceptable and as mentioned in the discussion, “Further analysis needs to be carried out to eliminate confounding factors and better understand the potential mechanisms of those associations”.

All that said, this is a large dataset, collected in a fairly unique manner. A resubmission could be considered, ideally focusing on some of the novelty in the data collection, and with a greater focus on descriptive statistics. If the deidentified data can be made available for an interactive visualization tool (eg. Shiny app), this might also be of interest, and could be discussed with the editorial team in advance.

Thank you for this comment. We added an additional descriptive table in the supplemental data and oriented our manuscript to highlight more of the novelty of our approach. We would be pleased to share deidentified aggregate data for an interactive visualization tool.
