# Supplementary material for: Acute respiratory distress syndrome after SARS-CoV-2 infection on young adult population: International observational federated study based on electronic health records through the 4CE consortium
Source: PLoS One. 2023 Jan 4;18(1):e0266985. doi: 10.1371/journal.pone.0266985 (PMC9812312; doi:10.1371/journal.pone.0266985)
Supplement: Attachment — Submitted filename: Response to Reviewers.docx [file pone.0266985.s007.docx]

**Response to reviewer**

RE: entitled Acute respiratory distress syndrome after SARS-CoV-2 infection on young adult population: international observational study based on electronic health records through the 4CE consortium

Dear Editor and Reviewer,

Thank you very much for your comments and constructive criticisms targeted at improving our manuscript submission. Below you will find our responses to the comments. The manuscript has also been modified accordingly.

Reviewer #1: The authors investigated the risk factors and outcomes of acute respiratory distress syndrome (ARDS) associated with SARS-CoV-2 infection in younger generations trough a federated analysis with large international cohorts. They found the poor outcome of ARDS associated with COVID-19 in younger generations and several diseases (chronic pulmonary disease, diabetes, obesity, paralysis, peptic ulcer) were risk factors for ARDS. The data were of valuable and importance, however, because of federated analysis (coding data) misunderstandings may happen from the results of the present study.

As stated in the Discussion, comorbidities and complications are confusing and misunderstanding. It is unknown that the high mortality rate at day 90 was due to COVID-19 phenotype or comorbidities in the cohort.

It is perfectly correct whereas all those patients have been diagnosed with ARDS. We add the following text to the discussion.

“Mortality at 30 days for ARDS patients of any age with COVID-19 was reported at 39%[[4]](https://www.zotero.org/google-docs/?teQWRo) and corresponds to the mortality for the older ARDS population in our study. The young ARDS population’s mortality at 90 days was smaller, around 16.2% with large variability between HS [11.2; 36.8%]. **Importantly, it was not possible to assess the attributable COVID-19 mortality from our data. “**

It is also unclear the management of ARDS in the nature of federated analysis. Were all patient treated in the ICU?

Identification of ICU admissions we not available. It appears that the accuracy of this information in EHR data was poor. It was particularly true at the beginning of the pandemic, where hallways were converted into ad hoc ICUs to support the surge of sick patients, without notification in the chart. This issue has already been discussed in a previous article from the consortium (4).

This missingness has been added to the limitation.

The use of respirator, HFNC, and/or ECMO should be demonstrated.

Through the 4CE consortium, the use of respirator, HFNC, and/or ECMO were not available. The collection of treatment data, described by specific codes in EHR, has proven to be too partial and largely heterogeneous between health system (even from the same country) to be collected.

This element has been added to the limitation.

“**Treatment like the use of mechanical ventilation, ECMO or even ICU admission were not collected. The collection of treatment data, described by specific codes in EHR, has proven to be too partial and largely heterogeneous between health systems (even from the same country) to be collected. It also appears that the accuracy of ICU admission in EHR data was poor. It was particularly true at the beginning of the pandemic, where hallways were converted into ad hoc ICUs to support the surge of sick patients, without notification in the chart. This issue has already been discussed in a previous article from the consortium** [**[17]**](https://www.zotero.org/google-docs/?bJFCBB).”

Coagulation disorder is the main pathogenesis in severe COVID-19. However, it is impossible to show the relationship between coagulation disorder and severity of COVID-19-ARDS from the coding data.

It is perfectly true, and we agree that EHR/coding data are not the correct tool to highlight this association and it was not the objective of the study.

**Data sharing**

The aggregate data would be shared. They will include for each center, the number and percentage of patients per age groups, per sex, per Elixhauser comorbidities, per complications for all groups.
