# Supplementary material for: Acute respiratory distress syndrome after SARS-CoV-2 infection on young adult population: International observational federated study based on electronic health records through the 4CE consortium
Source: PLoS One. 2023 Jan 4;18(1):e0266985. doi: 10.1371/journal.pone.0266985 (PMC9812312; doi:10.1371/journal.pone.0266985)
Supplement: Attachment — Submitted filename: Response to Reviewers.docx [file pone.0266985.s008.docx]

**Response to reviewer**

RE: entitled Acute respiratory distress syndrome after SARS-CoV-2 infection on young adult population: international observational study based on electronic health records through the 4CE consortium

Dear Editor and Reviewer,

Thank you very much for your comments and constructive criticisms targeted at improving our manuscript submission. Below you will find our responses to the comments. The manuscript has also been modified accordingly.

Reviewer #1: The authors responded well. I have no comment on the revised munuscprist including publication and research ethics.

Thank you

Reviewer #2: This study by Moal et al identifies risk factors associated with the development of ARDS following COVID-19 in young (18-49 years old) hospitalized adults. Further analysis compares this cohort with older (>49 years old) COVID-ARDS patients. They utilized electronic health records and federated (coded) analysis with multicentre international cohorts. Poor outcome of younger COVID-19-ARDS patients was associated with chronic pulmonary disease, diabetes, obesity, paralysis and peptic ulcers.

The way the data was collected makes it difficult to distinguish between comorbidities and complications. I am unsure why comorbidity data were not restricted to 1 year prior to diagnosis for the entire cohort.

Thanks for this remark which highlights a limit of our study. Indeed, the difficulty to distinguish between comorbidities and complications comes from the way data are collected. Comorbidities and complications are identified through ICD codes. In electronic health records, each code is attached to one specific hospitalization visit. For patients with prior hospitalizations, comorbidities are easily identified with the codes attached to those previous hospitalizations. However, for patient without previous hospitalization, the fact that electronic health records do not contain any code, does not mean that patient did not have comorbidities. For example, an obese patient without prior hospitalization would be identified as “obese“ only if we take into account the code associated with the index hospitalization.

For young ARDS patients, only 31% (312/1001) of them had a previous hospitalization.

This explains the fact that for our analysis, we use also the code related to the index hospitalization to identify comorbidities. To clarify this limit, we added in the discussion the following paragraph:

“A limitation of relying on billing codes to identify comorbidities is the challenge of accurately distinguishing comorbidities from complications. In our analysis, comorbidities were considered as those diagnoses from billing codes assigned up to one year before and up to 90 days after the admission. In electronic health records, each code is attached to one specific hospitalization visit. For patients with prior hospitalizations, comorbidities are easily identified with the codes attached to those previous hospitalization. However, for patient without previous hospitalization, the fact that electronic health records do not contains any code, do not mean that patient did not have comorbidities. For example, an obese patient without prior hospitalization would be identified as “obese“ only if we take into account the code associated with the index hospitalization ”.

Were the severe COVID-ARDS patients ventilated and could some of these patients have developed ventilator associated lung injury, independently of COVID-19? The definition of severe COVID-19 usually would include some indication of oxygen requirement (either face mask, CPAP or ventilator support). The authors definition of severe COVID-19 could be that they had severe disease along with a COVID-19 infection. Did patients have critical illnesses (e.g. severe trauma, sepsis, etc) that were the primary cause of ARDS?

As mentioned in the discussion, the treatment like the use of mechanical ventilation, ECMO, or even ICU admission were not collected. Regarding the relation between ARDS and COVID-19 infection, patients included in our analysis had positive reverse transcription PCR tests for SARS-CoV-2 infection 7 days before to 14 days after the date of admission. This inclusion criterion allows us to ensure that included patients had the COVID-19 infection at least at the beginning of the hospitalization. Even if it is not possible to establish a clear temporal relationship or causality between ARDS and COVID-19 with ICD codes, it would be extremely rare that the development of ARDS during the hospitalization of a COVID-19 positive patient had no relation to COVID-19 infection. It is possible that COVID-19 infection was not the primary cause of the ARDS but most likely had an impact on the ARDS development.

To clarify this point, we added in the discussion the following paragraph:

“Regarding the relation between ARDS and COVID-19 infection, patients included in our analysis had positive reverse transcription PCR tests for SARS-CoV-2 infection 7 days before to 14 days after the date of admission. This inclusion criterion allows us to ensure that included patients had the COVID-19 infection at least at the beginning of the hospitalization. Even if it is not possible to establish a clear temporal relationship or causality between ARDS and COVID-19 with ICD codes, it would be extremely rare that the development of ARDS during the hospitalization of a COVID-19 positive patient had no relation to COVID-19 infection. It is possible that COVID-19 infection was not the primary cause of the ARDS but most likely had an impact on the ARDS development.”

It seems that the most important comparator group (severe COVID with no ARDS) were removed from these analyses. This could have introduced biases comparing two extremes of disease (hospitalised mild disease vs severe COVID-ARDS). These analyses could simply be identifying risk factors associated with severe disease and not ARDS specifically.

As noticed, bias of selection could have been introduced because patients with mechanical ventilation, sedatives/anaesthetics or treatment for shock but without an ARDS code (SEVERE_NO_ARDS) were not included to identify comorbidities associated with ARDS.

The choice, to not include them in this comparison, was conducted to eliminate potential miscoded ARDS patients and patients with severe disease or care not related to SARS-CoV-2 infections but with a concomitant infection.

Those patients could have been included in the ARDS population, but the objective of this study was to focus precisely on ARDS patients, and this grouping would have resulted in a significant measurement bias. Especially because the number of young SEVERE_NO_ARDS patients is greater than the one of ARDS patients. Moreover, for the detection of ARDS patients, a correct sensibility is expected as billing code is related to reimbursement in most countries and ARDS is associated with heavy care.

Another option would have been to group those patients with the NO_SEVERE patients, but this option would also result in a significant measurement bias.

In addition, we believe that the descriptive analysis of the SEVERE_NO_ARDS brings credits to this choice (e-table 1). Indeed, the young SEVERE_NO_ARDS population had a higher percentage of women (SEVERE_NO_ARDS: 52%, ARDS: 33%, No SEVERE: 44%). They also had a higher percentage of patients with previous contact with the healthcare system (SEVERE_NO_ARDS: 72%, ARDS: 31%, No SEVERE: 57%). Moreover, 15.1% of the SEVERE_NO_ARDS patients had a billing code associated with pregnancy, 36.1% with long-term drug therapy. These results suggest that the COVID-19 infection was simply concomitant but not the main cause of these hospitalizations.

To clarify this point, we added in the discussion the following paragraph:

To identify comorbidities associated with ARDS following hospitalization with COVID, a comparison was performed considering only non severe patients. Patients with mechanical ventilation, sedatives/anesthetics, or treatment for shock but without ARDS code were not included, which could generate a selection bias. This choice was conducted to eliminate potential miscoded ARDS patients and patients with severe disease or care not related to SARS-CoV-2 infection but with a concomitant infection. Those patients could have been included in the ARDS population, but the objective of this study was to focus precisely on ARDS patients, and this grouping would have resulted in a significant measurement bias. Especially because the number of young SEVERE_NO_ARDS patients is greater than the one of ARDS patients. In addition, we believe that the descriptive analysis of the SEVERE_NO_ARDS brings credit to this choice (e-Table 1). Compared to the other groups, SEVERE_NO_ARDS population had the higher percentage of women (52.2%) and of patients with previous contact with the healthcare system (72%). In addition, 15.1% of those patients had a billing code associated with pregnancy and 36.1% with long-term drug therapy. These results suggest that the COVID-19 infection was simply concomitant but not the main cause of these hospitalizations.

I wonder whether the authors have split these analyses into smaller age ranges, i.e. 18−25, 26−49, 50−69, 70−79 and 80+ years old, or are the numbers per group too small. I wouldn’t class an individual of 49 years of age to be a young adult (compared to someone 50 years as old).

Through the 4CE consortium, the age is not collected as a numeric value but by age groups (18−25, 26−49, 50−69, 70−79, and 80+ years old), as described in the method section. It is an intrinsic limit of our analysis, but this choice has been made to bring more security/de-identification to the data collection process which permits an easier regulatory process for international aggregated data sharing.

To highlight this limit, we have added in the discussion the following sentences:

“More detailed analysis on age’s threshold was not possible because age was intentionally not collected by the 4CE consortium as a continuous variable. This choice was made to ensure greater security/de-identification on the data collection process which allowed for an easier regulatory process for international aggregated data sharing”

Was ARDS criteria the same for each site? i.e. as per Berlin criteria.

The criteria for ARDS detection were the same for each site; ARDS patients were detected with the ICD10 code J80 - Acute respiratory distress syndrome. In each healthcare system the order to code J80 was to meet the Berlin criteria.

Reviewer #3: Thank’s to the authors for this interesting piece of work. The difficulty of conducting an international survey was mentioned in this study by peer reviewers and authors. Misunderstandings remain about the methodology used for patient recruitment since there is no consensual health database on all health systems explored. The definition of the keywords searched in the electronic databases had to appear in the methodology.

No keywords were searched in the electronic databases. Comorbidities and complications were Identified with billing code. Each health system was using a common classification (ICD10) for those billing codes. The recruitment was based on the visits date and the presence of a positive PCR SARS-CoV-2 test**.**

Also, how did you discriminate hospitalizations whose causes were other than COVID-19?

The cause of hospitalization was not collected in the analysis. As explained in an answer to the reviewer 2, whereas it is not possible to establish a clear temporal relationship or causality between ARDS and COVID-19 with ICD codes, it would be extremely rare that the development of ARDS during the hospitalization of a COVID-19 positive patient had no relation with COVID-19 infection. It is possible that COVID-19 infection was not the primary cause of the ARDS but most likely had an impact on the ARDS development. For patients without ARDS, COVID-19 could only be a concomitant infection to the hospitalization linked to a different cause, which makes it a good control population

How do you justify the choice of a poorly frequented hospital in Germany? And what is the benefit of having included this health center when its presence does not influence the results of the study?

Thank you very much to notice this typo in the Table 1. The number of Inpatient in the German hospital was not 715 but 71500 patients. By mistake in this table, the numbers finishing by a 0, had lost their 0. The Table 1 has been corrected.
